# MicroRNAs and Sepsis-Induced Cardiac Dysfunction: A Systematic Review

**DOI:** 10.3390/ijms22010321

**Published:** 2020-12-30

**Authors:** Alice Chiara Manetti, Aniello Maiese, Marco Di Paolo, Alessandra De Matteis, Raffaele La Russa, Emanuela Turillazzi, Paola Frati, Vittorio Fineschi

**Affiliations:** 1 Department of Surgical Pathology, Medical, Molecular and Critical Area, Institute of Legal Medicine, University of Pisa, 56126 Pisa (PI), Italy; alicechiara812@gmail.com (A.C.M.); aniello.maiese@unipi.it (A.M.); marco.dipaolo@unipi.it (M.D.P.); emanuela.turillazzi@unipi.it (E.T.); 2 IRCSS Neuromed Mediterranean Neurological Institute, Via Atinense 18, 86077 Pozzilli (IS), Italy; raffaele.larussa@uniroma1.it (R.L.R.); paola.frati@uniroma1.it (P.F.); 3 Department of Anatomical, Histological, Forensic and Orthopaedic Sciences, Sapienza University of Rome, Viale Regina Elena 336, 00161 Rome (RM), Italy; alessandra.dematteis@uniroma1.it

**Keywords:** sepsis, cardiac dysfunction, microRNA, messenger RNA, long noncoding RNA

## Abstract

Sepsis is a severe condition characterized by systemic inflammation. One of the most involved organs in sepsis is the heart. On the other hand, heart failure and dysfunction are some of the most leading causes of death in septic patients. miRNAs are short single-strand non-coding ribonucleic acids involved in the regulation of gene expression on a post-transcriptional phase, which means they are a part of the epigenetic process. Recently, researchers have found that miRNA expression in tissues and blood differs depending on different conditions. Because of this property, their use as serum sepsis biomarkers has also been explored. A narrative review is carried out to gather and summarize what is known about miRNAs’ influence on cardiac dysfunction during sepsis. When reviewing the literature, we found at least 77 miRNAs involved in cardiac inflammation and dysfunction during sepsis. In the future, miRNAs may be used as early sepsis-induced cardiac dysfunction biomarkers or as new drug targets. This could help clinicians to early detect, prevent, and treat cardiac damage. The potential role of miRNAs as new diagnostic tools and therapeutic strategies worth deepening the complex network between non-coding RNA and biological pathways. Additional studies are needed to further investigate their role in sepsis-induced myocardium injury.

## 1. Introduction

Sepsis is a severe systemic inflammatory response (SIRS) associated with an infectious process [1]. It can progress to multiple organ dysfunction, which can lead, in turn, to septic shock [2,3]. Sepsis is a common condition characterized by a high mortality rate [4], especially in patients with comorbidities [5]. Cardiac involvement is one of the main causes of death in sepsis [6,7,8]. Moreover, hospitalization and the treatment of sepsis represent an important expenditure for healthcare systems in developed countries [9]. Therefore, sepsis could be considered as a healthcare concern, and a lot of efforts must be spent in developing adequate therapies. Even though a lot of studies have been carried out, the pathogenesis and the pathophysiology of organ involvement in sepsis is still not completely clear. To understand the underlying causes of the cardiovascular dysfunction which occurs in sepsis, the interaction between various immunosuppressive and pro-inflammatory pathways has been thoroughly studied [10]. In this tangled combination of factors, the role of microRNA (miRNA or miR) has recently been considered.

MiRNAs are short single-strand non-coding ribonucleic acids involved in the regulation of gene expression on a post-transcriptional phase, which means they are a part of the epigenetic process [11,12]. Particularly, miRNAs target the 3′untranslated region (UTR) of specific messenger RNA (mRNAs), and therefore, they prevent mRNA translation [13]. Besides, through the interaction with the AU-rich elements (AREs), under certain circumstances miRNAs can also up-regulate protein translation [14]. MiRNAs are involved in the pathophysiology of various diseases [15]. Since miRNAs play a role in the regulation of numerous post-transcriptional pathways involved in the inflammatory response, their influence on the sepsis process has been investigated [16,17,18,19]. MiRNAs are produced in cells but they can also be found as a stable molecule in plasma and other bodily fluids (extracellular miRNAs) [20]. Because of this property, miRNAs could be used as serum sepsis biomarkers [21,22,23,24,25,26,27,28,29,30]. It could be interesting to evaluate if specific miRNAs are involved in the myocardial inflammation underlying heart failure in patients with sepsis. Furthermore, understanding all the pathophysiologic mechanisms involved in cardiac damage could provide the chance to develop new therapies.

Therefore, a review of the currently available data about miRNAs involved in cardiac damage in septic systemic inflammatory response has been carried out.

## 2. Materials and Methods

The present systematic review was carried out according to the Preferred Reporting Items for Systematic Review (PRISMA) standards [31]. A systematic literature search and a critical appraisal of the collected studies was conducted. An electronic search of PubMed, Science Direct Scopus, Google Scholar, and Excerpta Medica Database (EMBASE) from the inception of these databases to August 2020 was performed. The search terms were “microRNA + sepsis induced cardiac dysfunction”, “microRNA + sepsis-induced cardiac dysfunction”, “miRNA + sepsis induced cardiac dysfunction”, “miRNA + sepsis-induced cardiac dysfunction”. The bibliographies of all located papers were examined and cross-referenced for further relevant literature. The methodological appraisal of each study was conducted according to the PRISMA standards, including the evaluation of bias. Data collection entailed study selection and data extraction. Three researchers (R.L.R., P.F., M.D.P.) independently examined the papers with title or abstracts that appeared to be relevant and selected the ones that analyzed the miRNA used to identify cardiac dysfunction in sepsis-related death. Disagreements concerning eligibility between the researchers were resolved by a consensus process. No unpublished, pre-print, or grey literature was searched. Only papers in English were included in the search. Data extraction was performed by two investigators (A.M., A.C.M.) and verified by two other investigators (V.F., E.T.). This study was exempt from institutional review board approval as it did not involve human subjects.

## 3. Results

An appraisal based on titles and abstracts as well as a hand search of reference lists were carried out. The reference lists of all located articles were reviewed to detect still unidentified literature. This search identified 152 articles, which were then screened based on their abstract. The resulting 152 references were screened to exclude duplicates, which left 109 articles for further consideration. In addition, papers not written in English were excluded, and the following inclusion criteria were used: (1) original research articles, (2) reviews and mini-reviews, and (3) case report/series. These publications were carefully evaluated, taking into account the main aims of the review. This evaluation left 35 original research articles.

**Figure 1:**
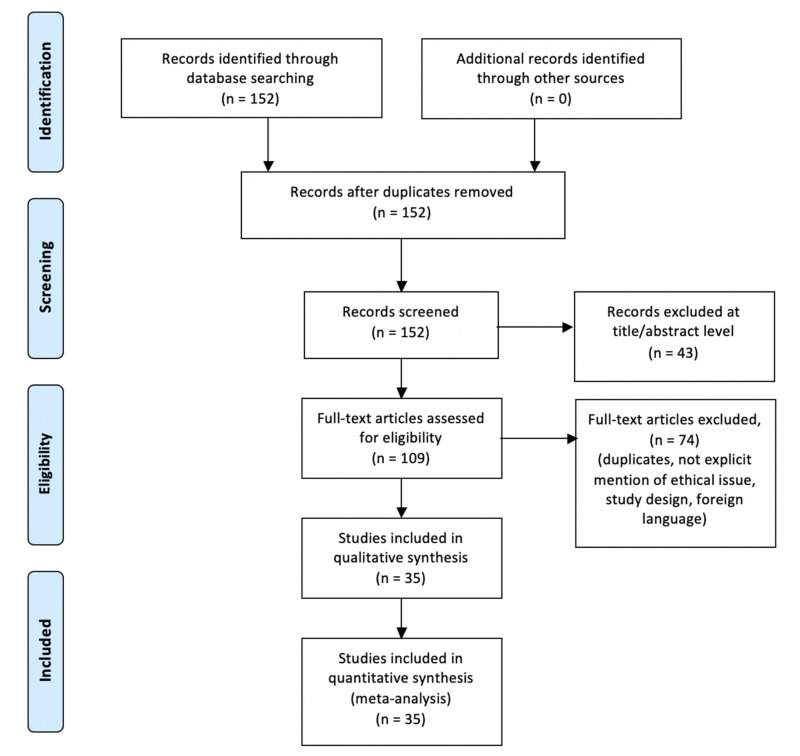
Methodology search strategy: we identified 152 articles, the screening based on their abstract left 109 studies and after a careful evaluation based on the aims of this review 35 research articles were included.

Figure 1 illustrates our search strategy. Table 1 shows the scientific papers included in the review.

### miRNA Expression in Sepsis-Related Cardiac Dysfunction

MiRNAs sequences are entirely known and well preserved through species. Immunosuppression, pro-inflammation with these are also important pathways in understanding the role of miRNAs in septic myocardial injury. At the moment, there are few studies investigated lncRNAs-miRNAs interaction in sepsis and cardiac damage. The potential role of miRNAs as new diagnostic tools and therapeutic strategies worth deepening the complex network between non-coding RNA and biological pathways.

Wang et al. evaluated the miRNA expression profiles in myocardial tissue in the cecal ligation and puncture (CLP) mouse model [32]. They reported that miR-223 was downregulated after severe CLP surgery, with an increase of inflammatory response and myocardial depression. Some clinical studies supported these findings, showing a reduction of serum miR-223 in patients who died of sepsis [33,34]. It is noteworthy that a decrease in seric level of miR-223 was also linked with cardiac mortality in chronic kidney disease patients [35]. It seemed that miR-223-5p inhibits the translation of sempahorin3A. A multi-omics study also demonstrated that MiR-223 has a profound role on the NF-κB system and it is involved in inflammation regulation (i.e., monocyte differentiation) [36].

Xue et al. showed that miR-27a expression is elevated in lipopolysaccharide (LPS) exposed mice’s myocardium [37]. They also conducted in vitro experiments to understand its mechanism of action: it seemed that miR-27a regulates the nuclear factor (erythroid-derived)-like 2 (Nrf2) expression. Nrf2 is a transcription factor that regulates the expression of antioxidant enzymes [38]. In 2015, Gao et al. demonstrated that an elevation of miR-146a expression attenuates myocardial dysfunction in polymicrobial sepsis by inhibiting NF-kB activation [39]. In 2018, An et al. confirmed through an in vitro experiment the attenuation of inflammation induced by miR-146a [40]. They also found a correlation between miR-146a elevation and an enhanced ErbB4 expression. More recently, Xie et al. corroborated these findings, suggesting miR-146a could influence the TLR-4/NFκB signaling pathway [41]. Wang et al. proved that also miR-146b plays a protective role against sepsis-induced myocardial damage [42]. They suggested it works via inhibition of Notch1, which is involved in the development of heart and in the inflammatory response.

In another work, Ma et al. demonstrated a similar protective effect obtain by increasing the expression of miR-125b [43]. Chen et al. showed that miR-125b decreases in the CLP mouse model [44]. Moreover, when a miR-125b mimic was transfected, the cardiac function improved. Furthermore, they investigated the role of the long noncoding RNA (lncRNA) MALAT1. They concluded that there is a correlation between MALAT1, miR-125b, and the p38 MAPK/NFκB pathway, and therefore MALAT1 enhances the myocardial inflammation. MALAT1 was also the subject of the work of Wei and Liu [45]. They found that miR-150-5p is inhibited by MALAT1 and therefore they have opposite roles in the sepsis-induced myocardial inflammation. While the overexpression of miR-150-5p is protective, the overexpression of MALAT1 worsens cardiac inflammation. They suggested MALAT1 is a miR-150-5p inhibitor. It also seemed that miR-150-5p regulates the NF-kB pathway. A more recent study confirmed the protective effect of miR150-5p on myocardiocytes in sepsis [46].

In two different studies, Wang et al. investigated the changes in miR-21-3p and miR-155 expression in the cardiac tissue of mice exposed to LPS [47,48]. Both miR-21-3p and miR-155 were up-regulated in the myocardial tissue after the intraperitoneal injection of LPS. Indeed, the administration of their relative antagomiRNA (antagomiR) before the LPS exposure led to a diminishing of the cardiac dysfunction, and vice versa the previous administration of the agomiRNA (agomiR) worsened the cardiac dysfunction. To evaluate the clinical relevance of their findings, they also measured miR-21-3p in the blood of septic patients with cardiac involvement, revealing that it was greater than in septic patients without cardiac dysfunction. Diao and Sun conducted a similar work [49]. They evaluated the expression of miR-124a in LPS-induced sepsis mice’s myocardium and its variation following the administration of its antagomiR and agomiR. MiR-124a was down-regulated in septic mice while its inhibition and stimulation showed respectively a worsening and improvement of the cardiac function.

Very interesting work was carried out by Zheng et al. [50]. As first, they measured the level of miR-135a in the serum of patients with sepsis-induced cardiac depression, finding a correlation between the level of miR-135a and the severity of the myocardial dysfunction. Then, they performed CLP surgery in miR-135a-transfected mice. Myocardial inflammation in transfected mice was higher than in non-transfected, while the cardiac function was decreased. They also suggested that the miR-135a pro-inflammatory effect could be mediated by the activation of the p38 MAPK/NF-κB pathway.

The injection of miR-155 mimic after CLP surgery in mice was found to be protective against cardiac dysfunction by Zhou et al., as well as the previous transfection of miR-155 diminished the inflammatory cell infiltration into the myocardium [51]. The authors also suggested that miR-155 inhibits the expression of β-arrestin 2 (Arrb2), which is a protein involved in immune system regulation. Other authors demonstrated the up-regulation of miR-214 during sepsis in mice [52]. In their study, myocardial inflammation, apoptosis, and dysfunction were decreased when the miR-214 expression was enhanced via its precursor, and, vice versa, they were worsened by its inhibitor. In a different study, it was demonstrated that miR-214-3p inhibits autophagy via the PTEN/AKT/mTOR pathway [53].

A study of the miR-874 expression was conducted by Fang et al. [54]. They found that miR-874 was up-regulated in sepsis patients’ serum, in LPS-induced sepsis mice, and septic mice’s myocardiocytes. They found a negative correlation between miR-874 and lncRNA H19 and aquaporin 1 (AQP1), suggesting that H19 inhibits miR-874 expression, which in turn inhibits AQP1 expression. In previous studies, AQP1 was demonstrated to be involved in other pathophysiologic mechanisms, such as tumor development, inflammatory cytokines release (via NF-Κb signaling pathway), and polycystic kidney disease (via Wnt signaling pathway) [55,56,57].

MiR-93-3p was down-regulated in LPS-treated cells in Tang et al.’s work [58]. It seemed to be involved in the regulation of toll-like receptor 4 (TRL4) translation. The overexpression of miR-93-3p repressed apoptosis and cytokine expression, suggesting a protective role against septic-induced cardiac damage. Another miRNA’s function was studied by Yao et al. [59]. They found that miR-25, which is decreased during sepsis, if overexpressed had got a protective effect against apoptosis in LPS-induced cell damage. They suggested miR-25 influences the TLR4/NF-kB pathway and directly targets the phosphatase and tensin homolog (PTEN).

Wu et al. found that also miR-494-3p targeted PTEN [60]. miR-494-3p is down-regulated in septic patients’ blood and its decrease correlates with the cardiac dysfunction. MiR-494-3p up-regulation protects rat cardiomyocyte against apoptosis. MiR-23b was found to be elevated in CLP mice’s myocardium by Zhang et al. [61]. Its inhibition not only reduced myocardial dysfunction but also attenuated cardiac remodeling. The authors stated that the miR-23b′s target gene is the 5′TG3′-interacting factor 1 (TGIF1), which in turn inhibits the transforming growth factor β1 (TGF- β1), known to be involved in fibrogenesis [62,63]. It must be added, however, that Cao et al. reported opposite results [64]. In their study, miR-23b seemed to attenuate sepsis-induced cardiac dysfunction. They up-regulated miR-23b in cardiomyocytes, both in vivo and in vitro, and then measured the cardiac function and the inflammatory cytokine secretion. They were respectively increased and decreased. There was also a reduction in adhesion molecules expression, NF-κB pathway activation, and caspase-3 activity. Unfortunately, Cao et al. did not explore the reasons behind this difference. Maybe it is reasonable to hypothesize different pathways for the two strands of the same pri-miRNA.

MiR-495 was found to be down-regulated in blood samples of septic patients by Guo et al. [65]. Furthermore, it was more decreased in patients who developed septic shock. The authors also created a rat sepsis-model through CLP modelling. Septic rats’ myocardium and serum disclosed a decrease in MiR-495. The cardiac function was impaired as well. The injection of agomiR-495 reduced inflammation and reversed the myocardial dysfunction. Zhu et al. [66] investigated the role of miR-98 in sepsis-cardiac dysfunction. MiR-98 was down-regulated in the myocardium of CLP-mice. Mice who received the injection of the agomiR-98 showed an increase in cardiac function, less myocardial damage and apoptosis (via Cleaved caspase-3 and Bax protein’s inhibition), and a different pattern of cytokines. Specifically, tumor necrosis factor α (TNF- α) and interleukine-6 (IL-6) were increased, while IL-10 was decreased. Another study showed that miR-208a-5p is elevated in the myocardium during sepsis and its inhibition could reduce the myocardial damage induced by inflammation, probably by influencing the NF-kB/HIF-1α signaling pathway [67].

Sun et al. recognized a positive correlation between miR-328 serum level and sepsis in human patients [68]. Moreover, miR-328 showed to be correlated with cardiac dysfunction in the CLP rat model, while the injection of its antagomiR seemed to reduce inflammation and ameliorate the impairment. In 2019, Zhang et al. identified 78 miRNAs expression of which changed during the septic state in rat heart [69]. They then constructed a complex network to represent the relationship between miRNAs and circular RNAs (circRNAs), a different kind of non-coding RNA involved in biological pathway control via miRNAs’ inhibition [70].

MiR-29a expression in sepsis appeared to be enhanced in a study recently conducted by Zhu et al. [71]. They evaluated the role of a lncRNA, CRNDE, in sepsis-induced myocardial damage. CRNDE diminished apoptosis, inflammation, and oxidative stress in cardiomyocytes treated with LPS via miR-29a inhibition. Contrasting results were provided by Song et al. [72]. In their study, miR-29a seemed to be down-regulated by LPS and it had a positive role in preventing and attenuating cardiac damage. The lncRNA CYTOR was studied by Chen et al. [73]. CYTOR regulates the expression of miR-24, which inhibits the translation of X-chromosome-linked inhibitor of apoptosis (XIAP). As a result of this pathway, down-regulation of CYTOR or up-regulation of miR-24 appeared to worsen sepsis-induced myocardial injury via activation of apoptosis. Sun et al. demonstrated the role played by lncRNA KCNQ1OT1 in the regulation of miR-192-5p in myocardium damage by sepsis [74]. KCNQ1OT1 downregulated miR.192-5p, which in turn inhibits the translation of XIAP, improving myocardiocyte viability and contrasting apoptosis.

Nuclear enriched abundant transcript 1 (NEAT1) is another lncRNA found to be involved in sepsis-induced myocardial damage in a recent study [75]. NEAT1 exerts a negative control on miR-144-3p. NEAT1 was found to be elevated in myocardial cells when LPS was administered, while obviously miR-144-3p was decreased. MiR-144-3p seemed to be involved in apoptosis and inflammation in myocardial cells via the NF-kB pathway. Xing et al. investigated the role of lncRNA myocardial infarction associated transcript (MIAT) in sepsis-induced myocardial injury [76]. MIAT appeared to be a down-regulator of miR-330-5p. From their study, seemed that miR-330-5p attenuates myocardial oxidative stress and inflammatory response, thanks to its target protein, tumor necrosis factor receptor-associated factor 6 (TRAF6), which is involved in NF-kB signaling. miR-330-5p is down-regulated in septic myocardiocytes.

In another work the interaction between the lncRNA component of mitochondrial RNA processing (RMRP) and miR-1-5p was evaluated [77]. Herein, the protective role of RMRP against myocardial sepsis-induced injury and mitochondrial damage was reported. RMRP inhibits miR-1-5p, which in turn targets the heat shock protein HSPA4 (previously known as hsp70).

## 4. Discussion

In the last few years, interest in how miRNAs influence various signaling pathways has increased [13,78,79,80]. In particular, their role in the pathophysiology of diseases has been the subject of many studies [15,81,82,83,84]. MiRNAs are known to influence the inflammation process [82,85,86]. Since sepsis is considered as a systemic and runaway inflammatory response to infectious diseases, the existence of numerous miRNAs involved in sepsis pathophysiology should not surprise [16,17,18,19,87,88]. Concerning the role of miRNAs in sepsis-induced cardiac dysfunction, a lot still has to be disclosed.

**Figure 2:**
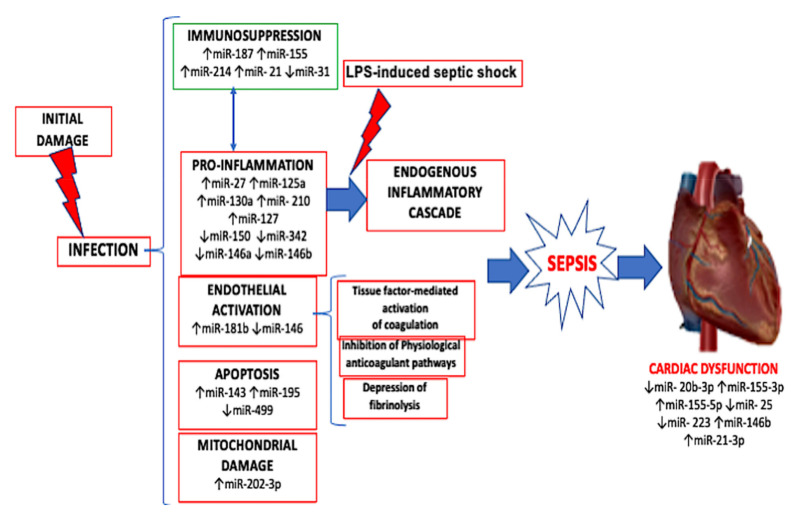
Mechanism of action of the activation of the major players of cardiac dysfunction induced by sepsis with up and down regulation of miRNAs involved in the mechanism of infection.

MiRNAs’ sequences are entirely known and well preserved through species. Furthermore, their synthesis is relatively easy [89]. These characteristics make miRNAs the perfect subject to study in order to find new diagnostic techniques and therapeutic strategies (Figure 2).

When reviewing the literature, we found at least 77 miRNAs involved in cardiac inflammation and dysfunction during sepsis, but we are sure we have just scratched the surface (Table 2).

MiR indicates microRNA; HSPA4, the heat shock protein previously known as hsp70; SORBS2, sorbin and SH3 domain containing 2; TGIF1, 5′TG3′-interacting factor 1; PTEN, phosphatase and tensin homolog; TRAF6, tumor necrosis factor receptor-associated factor 6; IκκB, inhibitor of nuclear factor kappa-B kinase subunit beta; XIAP, X-chromosome-linked inhibitor of apoptosis; Nrf2, nuclear factor (erythroid-derived 2)-like 2; SIRT1, sirtuin 1; TRL4, toll-like receptor 4; HMGA2, high mobility group at-hook 2; STX2, syntaxin-2; IRAK1, interleukin-1 receptor-associated kinase 1; ErbB4, erb-B2 receptor tyrosine kinase 4; Akt2, serine/threonine kinase 2; Pea15a, phosphoprotein enriched in astrocytes; Arrb2, β-arrestin 2; SOCS2, suppressor of cytokine signaling 2; Sema3A, semaphorin3A; AQP1, aquaporin 1.

The NF-kB family comprises numerous transcription factors that regulate various biological pathways. In particular, it is involved in the immune system response and in the pathogenesis of some malignancies [90,91]. NF-kB proteins are also implicated in sepsis and SIRS [92,93]. Therefore, it is not surprising that miRNAs implicated in sepsis myocardial damage regulate, directly or indirectly, some NF-kB-mediated pathways involved in cardiac dysfunction during sepsis [39,45,59,75,76]. Some studies suggest that inflammation and infections could have a role in atherosclerotic plaque development and coronary heart disease (CHD) [94,95]. Furthermore, a recent review summed up the main miRNAs involved in atherosclerosis [96]. It is interesting to notice that a lot of miRNAs are both involved in sepsis-induced cardiac dysfunction and atherosclerosis. In particular, miR-223, which regulates the NF-κB signaling pathway, seems to influence plaque formation and thrombosis inhibiting tissue factor expression [36,96]. These evidences could suggest a correlation between CHD, sepsis, and myocardial damage.

It could be interesting to also focus on miR-23b. One study demonstrated it is involved in myocardial fibrotic changes via targeting TGIF1 [61]. This could be a mechanism of late sepsis cardiac remodeling. Another protein, which was mentioned a few times in the present review, is PTEN. It is a phosphatase that regulates various cellular signaling pathways and has tumor suppression properties. Some of the aforementioned studies demonstrated that PTEN is targeted by miRNAs in sepsis-induced cardiac damage [53,59,60]. A further complication in understanding the role of miRNAs in septic myocardial injury is represented by lncRNAs, as evidenced by some research studies mentioned above [45,73,74]. Since they have the property of inhibiting miRNAs, they seem to play a crucial role in the tangled forest of those interconnected signaling pathways. At the moment, there are few studies investigated lncRNAs-miRNAs interaction in sepsis and cardiac damage.

## 5. Conclusions

In conclusion, even though recent studies have provided new insight in sepsis-induced cardiac dysfunction miRNAs involvement, the complete network of influences is still only partially understood. Sepsis is a challenge and a great expenditure for healthcare systems and sepsis-induced cardiac dysfunction is one of the major causes of death among septic patients. In the future, miRNAs may be used as early sepsis-induced cardiac dysfunction biomarkers or as new drug targets. This could help clinicians to early detect, prevent, and treat cardiac damage. The potential role of miRNAs as new diagnostic tools and therapeutic strategies worth deepening the complex network between non-coding RNA and biological pathways. Further clinical investigations are required to establish miRNAs’ role in diagnostic and therapeutic approaches in myocardial injury during sepsis.

## Figures and Tables

**Table 1:** Review of the literature on miRNA and cardiac dysfunction in sepsis. MiR indicates microRNA.

Author and Reference	Year of Publication	Sepsis Model	Brief Description of miRNAs in Sepsis-Induced Cardiac Injury
Wang et al. [32]	2014	In vivo	miR-223 (both 3p and 5p) down regulated, its absence enhances myocardial inflammation
Xue et al. [37]	2015	In vivo + in vitro	miR-27a up regulated, its overexpression enhances myocardial inflammation
Gao et al. [39]	2015	In vivo + in vitro	miR146a up regulated, its overexpression attenuates myocardial disfunction
Ma et al. [43]	2016	In vivo + in vitro	miR-125b down regulated, its overexpression attenuates myocardial disfunction
Wang et al. [47]	2016	In vivo + in vitro	miR-21-3p up regulated, its down regulation attenuates myocardial inflammation, while its overexpression worsens it.
Wang et al. [48]	2016	In vivo	miR-155 up regulated, its inhibition attenuates myocardial apoptosis, its overexpression worsens it.
Diao et al. [49]	2017	In vivo + in vitro	miR-124a down regulated, its overexpression attenuates myocardial damage, its down regulation worsens it.
Zheng et al. [50]	2017	In vivo + in vitro	MiR-135a up regulated, its overexpression worsens myocardial inflammation
Zhou et al. [51]	2017	In vivo	MiR-155 overexpression attenuates myocardial damage
An et al. [40]	2018	In vitro	miR-146a up regulated, its overexpression attenuates myocardial inflammation
Chen et al. [44]	2018	In vivo + in vitro	MiR-125b down regulated, its overexpression attenuates myocardial damage
Ge et al. [52]	2018	In vivo	MiR-214 up regulated, its overexpression attenuates myocardial apoptosis and damage
Fang et al. [54]	2018	In vivo + in vitro	MiR-874 up regulated, its inhibition attenuates myocardial dysfunction
Wang et al. [42]	2018	In vivo + in vitro	MiR-146b up regulated, its overexpression attenuates myocardial inflammation
Zhang et al. [61]	2018	In vivo + in vitro	MiR-23b up regulated, its inhibition attenuates myocardial dysfunction and fibrosis;
Tang et al. [58]	2018	In vitro	MiR-93-3p down regulated, its overexpression attenuates inflammation and apoptosis
Yao et al. [59]	2018	In vivo + in vitro	MiR-25 down regulated, its overexpression attenuates apoptosis
Wu et al. [60]	2018	In vivo + in vitro	MiR-494-3p down regulated and correlated with myocardial damage, its overexpression attenuates myocardial injury and apoptosis
Cao et al. [64]	2019	In vivo + in vitro	MiR-23b up regulated, its overexpression attenuates myocardial inflammation and apoptosis
Guo et al. [65]	2019	In vivo	MiR-495 down regulated, its overexpression attenuates myocardial dysfunction
Xie et al. [41]	2019	In vivo	MiR-146a up regulated, its overexpression attenuates myocardial inflammation and apoptosis
Zhu et al. [66]	2019	In vivo	MiR-98 down regulated, its overexpression attenuates myocardial damage, apoptosis, and inflammation
Zhang et al. [69]	2019	In vivo	78 miRNAs differently expressed in myocardium during sepsis
Wei et Liu [45]	2019	In vitro	MiR-150-5p down regulated, its overexpression attenuates inflammation
Chen et al. [73]	2020	In vivo + in vitro	MiR-24 up regulated, its inhibition attenuates myocardial apoptosis
Sun et al. [74]	2020	In vivo + in vitro	MiR-192-5p up regulated, its inhibition attenuates myocardial apoptosis
Ouyang et al. [67]	2020	In vivo	MiR-208a-5p up regulated, its inhibition attenuates myocardial damage
Sun et al. [68]	2020	In vivo	MiR-328 up regulated, its inhibition attenuates myocardial inflammation
Zhu et al. [46]	2020	In vivo + in vitro	MiR-150-5p down regulated, its overexpression attenuates myocardial apoptosis
Zhu et al. [71]	2020	In vivo + in vitro	MiR29a up regulated, its inhibition attenuates myocardial damage
Wei et al. [75]	2020	In vitro	MiR-144-3p down regulated, its overexpression attenuates myocardial damage
Xing et al. [76]	2020	In vivo + in vitro	MiR-330-5p down regulated, its overexpression attenuates inflammation and oxidative stress
Han et al. [77]	2020	In vivo + in vitro	MiR-1-5p enhances myocardial damage
Song et al. [72]	2020	In vivo + in vitro	MiR-29a attenuates myocardial damage
Sang et al. [53]	2020	In vivo	MiR-214-3p up regulated, its overexpression attenuates myocardial damage and autophagy

**Table 2:** MiRNAs involved in sepsis-induced cardiac dysfunction.

MiRNA	Expression in Sepsis (Myocardium and/or Serum)	Target Genes	Reference
MiR-1-5p	its inhibition is protective	*HSPA4*	Han et al. 2020 [77]
MiR-7a-5p	↑	*-*	Zhang et al. 2019 [69]
MiR-20b-3p	↓	*-*	Zhang et al. 2019 [69]
MiR-21-3p	↑ Its up-regulation worsens inflammation	*SORBS2*	Wang et al. 2016 [47]
MiR-21-5p			Zhang et al. 2019 [69]
MiR-23b	↑ its inhibition is protective	*TGIF1* *PTEN* *TRAF6* *IκκB*	Zhang et al. 2018 [61]
	its up-regulation is protective		Cao et al. 2019 [64]
MiR-24	↑ its inhibition is protective	*XIAP*	Chen et al. 2020 [73]
MiR-24-1-5p	↑	*-*	Zhang et al. 2019 [69]
MiR-24-2-5p	↑	*-*	Zhang et al. 2019 [69]
MiR-24-3p	↑	*-*	Zhang et al. 2019 [69]
MiR-25	↓ its up-regulation is protective	*PTEN*	Yao et al. 2018 [59]
MiR-27a	↑	*Nrf2*	Xue et al. 2015 [37]
MiR-27a-5p		*-*	Zhang et al. 2019 [69]
MiR-29a	↑ its inhibition is protective	*SIRT1*	Zhu et al. 2020 [71]
	↓ its up-regulation is protective		Song et al. 2020 [72]
MiR-30c-5p	↓	*-*	Zhang et al. 2019 [69]
MiR-30d-3p	↓	*-*	Zhang et al. 2019 [69]
MiR-92a-1-5p	↑	*-*	Zhang et al. 2019 [69]
MiR-93-5p	↓	*-*	Zhang et al. 2019 [69]
MiR-93-3p	↓ its up-regulation is protective	*TLR4*	Tang et al. 2018 [58]
MiR-98	↓ its up-regulation is protective	*HMGA2*	Zhu et al. 2019 [66]
MiR-99a-5p	↑	*-*	Zhang et al. 2019 [69]
MiR-122-5p	↑	*-*	Zhang et al. 2019 [69]
MiR-124a	↓ its up-regulation is protective	*STX2*	Diao et al. 2017 [49]
MiR-125b	↓ its up-regulation is protective	*TRAF6*	Ma et al. 2016 [43]
			Chen et al. 2018 [44]
MiR-126a-3p	↑	*-*	Zhang et al. 2019 [69]
MiR-128-3p	↑	*-*	Zhang et al. 2019 [69]
MiR-132-5p/3p	↑	*-*	Zhang et al. 2019 [69]
MiR-133a-3p/5p	↓	*-*	Zhang et al. 2019 [69]
MiR-135a	↑ Its up-regulation worsens inflammation	*-*	Zheng et al. 2017 [50]
MiR-143-3p	↑	*-*	Zhang et al. 2019 [69]
MiR-144-3p	↓ its up-regulation is protective	*-*	Wei et al. 2020 [75]
MiR-145-5p	↓	*-*	Zhang et al. 2019 [69]
MiR-146a	↑ its up-regulation is protective	*IRAK1* *TRAF6* *ErbB4*	Gao et al. 2015 [39]
			An et al. 2018 [40]
			Xie et al. 2019 [41]
MiR-146b	↑ its up-regulation is protective	*Notch1*	Wang et al. 2018 [42]
MiR-146b-5p/-3p			Zhang et al. 2019 [69]
MiR150-5p	↓ its up-regulation is protective	*Akt2*	Wei et Liu 2019 [45]
			Zhu et al. 2020 [46]
MiR-150-3p	↓		Zhang et al. 2019 [69]
MiR-155	↑ its up-regulation is protective	*Pea15a* *Arrb2*	Wang et al. 2016 [48]
			Zhou et al. 2017 [51]
MiR-155-3p	↑	*-*	Zhang et al. 2019 [69]
MiR-181b-1-3p	↑	*-*	Zhang et al. 2019 [69]
MiR-181b-5p	↓	*-*	Zhang et al. 2019 [69]
MiR-192-5p	↑ its inhibition is protective	*XIAP*	Sun et al. 2020 [74]
MiR-195-3p	↑	*-*	Zhang et al. 2019 [69]
MiR-200a-5p	↑	*-*	Zhang et al. 2019 [69]
MiR-201-5p	↓	*-*	Zhang et al. 2019 [69]
MiR-208a-5p	↑ its inhibition is protective	*SOCS2*	Ouyang et al. 2020 [67]
MiR-210-3p	↓	*-*	Zhang et al. 2019 [69]
MiR-214	↑ its up-regulation is protective	*PTEN*	Ge et al. 2018 [52]
MiR-214-3p			Sang et al. 2020 [53]
MiR-218a-5p	↑	*-*	Zhang et al. 2019 [69]
MiR-219a-1-3p	↑	*-*	Zhang et al. 2019 [69]
MiR-223-5p	↓	*Sema3A*	Wang et al. 2014 [32]
MiR-233-3p	↑	*-*	Zhang et al. 2019 [69]
MiR-233-5p	↑	*-*	Zhang et al. 2019 [69]
MiR-322-5p	↓	*-*	Zhang et al. 2019 [69]
MiR-328	↑ its inhibition is protective	*-*	Sun et al. 2020 [68]
MiR-330-5p	↑	*-*	Zhang et al. 2019 [69]
	↓ its up-regulation is protective	*TRAF6*	Xing et al. 2020 [74]
MiR-339-3p	↑	*-*	Zhang et al. 2019 [69]
MiR-340-3p	↑	*-*	Zhang et al. 2019 [69]
MiR-362-5p	↓	*-*	Zhang et al. 2019 [69]
MiR-369-5p	↑	*-*	Zhang et al. 2019 [69]
MiR-378a-5p	↓	*-*	Zhang et al. 2019 [69]
MiR-379-5p	↑	*-*	Zhang et al. 2019 [69]
MiR-380-3p	↑	*-*	Zhang et al. 2019 [69]
MiR-409a-3p	↑	*-*	Zhang et al. 2019 [69]
MiR-425-3p	↓	*-*	Zhang et al. 2019 [69]
MiR-434-5p	↑	*-*	Zhang et al. 2019 [69]
MiR-466b-5p	↑	*-*	Zhang et al. 2019 [69]
MiR-490-5p	↑	*-*	Zhang et al. 2019 [69]
MiR-494-3p	↓ its up-regulation is protective	*PTEN*	Wu et al. 2018 [60]
MiR-495	↓ its up-regulation is protective	*-*	Guo et al. 2019 [65]
MiR-503-5p	↓	*-*	Zhang et al. 2019 [69]
MiR-674-3p	↑	*-*	Zhang et al. 2019 [69]
MiR-708-3p	↑	*-*	Zhang et al. 2019 [69]
MiR-708-5p	↓	*-*	
MiR-874	↑ its inhibition is protective	*AQP1*	Fang et al. 2018 [54]
MiR-3557-3p	↓	*-*	Zhang et al. 2019 [69]

## Data Availability

No new data were created or analyzed in this study. Data sharing is not applicable to this article.
